# Are punitive parenting and stressful life events environmental risk factors for obsessive-compulsive symptoms in youth? A longitudinal twin study

**DOI:** 10.1016/j.eurpsy.2018.11.004

**Published:** 2019-02

**Authors:** G.C. Krebs, L.J. Hannigan, A.M. Gregory, F.V. Rijsdijk, B. Maughan, T.C. Eley

**Affiliations:** a King’s College London, MRC Social, Genetic and Developmental Psychiatry Centre, Institute of Psychiatry, Psychology & Neuroscience, De Crespigny Park, London, UK; b National and Specialist OCD and Related Disorders Clinic for Young People, South London and Maudsley NHS Foundation Trust, London, UK; c Institute of Health and Wellbeing, University of Glasgow, UK; d Department of Psychology, Goldsmiths, University of London, UK

**Keywords:** Obsessive-compulsive disorder, Stressful life events, Parenting, Adolescence, Etiology, Genetics

## Abstract

**Background:**

Punitive parenting and stressful life events are associated with obsessive-compulsive symptoms (OCS). However, the lack of longitudinal, genetically-informative studies means it remains unclear whether these factors represent environmentally-mediated risks for the development of OCS.

**Methods:**

Twins and siblings from the Genesis1219 study completed self-report questionnaires two years apart (Time 1: *N* = 2616, mean age = 15.0; Time 2: *N* = 1579, mean age = 17.0 years) assessing OCS, maternal and paternal punitive parenting, and dependent stressful life events. Multiple regression models tested cross-sectional and longitudinal associations between the putative environmental risk factors and obsessive-compulsive symptoms using: (a) individual scores; and (b) monozygotic twin difference scores. The aetiologies of significant phenotypic associations between putative risk factors and OCS were further examined using multivariate genetic models.

**Results:**

At a phenotypic level, maternal and paternal punitive parenting and stressful life events were all associated with OCS both cross-sectionally and longitudinally. However, only stressful life events predicted the subsequent development of OCS, after controlling for earlier symptoms. Genetic models indicated that the association between life events and change in OCS symptoms was due to both genetic (48%) and environmental (52%) influences. Overall, life events associated with *change* in OCS accounted for 1.2% of variation in OCS at Time 2.

**Conclusions:**

Stressful life events, but not punitive parenting, predict OCS change during adolescence at a phenotypic level. This association exists above and beyond genetic confounding, consistent with the hypothesis that stressful life events play a causal role in the development of obsessive-compulsive symptoms.

## 1 Introduction

It is well-established that obsessive-compulsive disorder (OCD) is influenced by genetic factors, with twin studies suggesting they account for approximately 45–65% of the variance in OCD symptoms in youth [1]. As well as demonstrating genetic influence on OCD, these studies also highlight the importance of the environment, particularly child-specific or ‘non-shared’ environmental factors [1]. However, surprisingly little is known about the specific aspects of the non-shared environment involved in OCD. Indeed, a recent systematic review concluded that no environmental risk factors for OCD have been compellingly demonstrated, and emphasised the need for longitudinal, population-based, genetically-informative studies [2].

Two putative environmental risk factors for OCD are maladaptive parenting and stressful life events [2]. OCD is associated with maladaptive parenting, particularly overprotection and rejection [[2], [3], [4]]. However, most studies have utilised retrospective report of childhood parenting experiences in adults with OCD, permitting possible recall bias. Furthermore, overreliance on cross-sectional data means that the direction of effects remains unclear. While parenting may influence offspring OCD, child behaviours may also shape parenting [5,6]. For example, parenting practices have been shown to be affected by child anxiety [[7], [8], [9]]. Finally, associations may arise as a result of the same genes influencing parenting behaviours in adults and the development of OCD symptoms in children [5,10]. Transmission of these genes from parents to children would give rise to an association between the two that reflects genetic rather than environmental risk.

Stressful life events have also been linked to OCD [2]. Again, most studies have been cross-sectional and used retrospective assessment. The only two longitudinal studies to date found prospective associations between life events and later OCD [11,12]. However, life events are also known to be influenced by genetic factors [7], which could thus confound the association with OCD. Only two studies of life events and OCD have attempted to control for genetic confounding [13,14]. Both studies used a within-identical twin pair design to control for familial effects, and identified associations between obsessive-compulsive symptoms (OCS) and retrospectively reported abuse and family disruption [13,14]. These studies provide preliminary evidence that certain stressful life events may be associated with OCS independent of familial effects, raising the possibility that they may be ‘true’ environmental risk factors. However, both studies were cross-sectional and used retrospective reports of childhood events.

In summary, maladaptive parenting and stressful life events appear to be associated with OCS, but most previous studies have relied on cross-sectional and retrospective designs, and few are genetically-informative. Thus, it remains unclear whether these factors represent true environmental risks for the development of OCS. The current study used a longitudinal, genetically-sensitive design to examine the relationship between maternal and paternal punitive discipline, stressful life events, and OCS during adolescence. The study had two broad aims. First, we tested whether punitive discipline and stressful life events were associated with OCS in adolescence at a phenotypic level. We hypothesized that punitive parenting and stressful life events would independently predict OCS both cross-sectionally and longitudinally. Second, we examined whether these associations remained significant after controlling for familial factors, and sought to directly estimate the aetiological influences mediating these associations. We hypothesized that punitive parenting and stressful life events would remain significant predictors of OCS even after accounting for familial confounds.

## 2 Methods

### 2.1 Participants

Participants were taken from the Genesis1219 project (G1219), a longitudinal study of twins and siblings. Recruitment and selection procedures have been described previously [15]. Ethical approval was given by the Institute of Psychiatry and South London and Maudsley NHS Trust Research Ethics Committee. Informed consent was obtained from parents of adolescents under 16 years and participants over 16. Twin zygosity was determined using parental ratings of physical similarity across two time-points [16].

The current study included data from waves 2 and 3, hereafter referred to as Time 1 and Time 2 respectively. An OCS measure was completed by 2616 participants at Time 1 (689 MZ twins, 1267 dizygotic (DZ) twins, 576 full siblings (FS), and 84 unknown) and 1579 participants at Time 2 (439 MZ twins, 816 DZ twins, 299 FS and 25 unknown). The mean age was 15.0 years (SD = 1.35; range = 13–17) and 17.0 years (SD = 1.17; range = 15–19), and the sample was 56% and 60% female at Times 1 and 2 respectively. Attrition was predicted by being male, being older, lower socioeconomic status, lower OCS at Time 1, and more stressful life events at Time 1 (see Table A1 in Appendix A). Of note, the magnitude of difference in scores between those who did and did not remain in the study was small for OCS (*M* = 3.86 versus 3.77) and stressful life events (*M* = 1.13 versus 1.36).

### 2.2 Measures

#### 2.2.1 Obsessive-compulsive symptoms

OCS was measured at Time 1 and 2 using the self-reported, six-item OCS subscale of the Spence Children’s Anxiety Scale [17]. Items assess a range of compulsions (e.g. checking, repeating, mental rituals) and obsessions (e.g. fear of negative events, experiencing negative mental pictures) and are scored on a 4-point scale, yielding a total between 0 and 18. The subscale has good internal consistency and test–retest reliability (Spence, 1998) and correlates well with the Children’s Yale-Brown Obsessive-Compulsive Scale, a clinician-administered measure of OCD severity [18]. Internal consistency in our sample was high (Cronbach’s alpha = .76 and 0.77 at Time 1 and 2, respectively).

#### 2.2.2 Punitive parenting

Parental punitive discipline was assessed at Time 1 using an adolescent-report, five-item measure of negative sanctions [10,19,20], adapted from a previously well-validated parent–child relationship measure [21]. Items assess the frequency with which punitive discipline methods are utilized (e.g. yelling at or ridiculing the child) and are scored on a 5-point scale, giving a total 0 and 20. The punitive discipline measure was completed by adolescents with respect to mothers and fathers separately; Cronbach’s alpha was 0.68 and .74, respectively.

#### 2.2.3 Stressful life events

Adolescents completed the Life Event Scale for Adolescents [22] at both time-points. This measure includes 12 “dependent” stressful events, which are events that can be influenced by the individual’s behaviour (e.g. breaking up with a boyfriend or girlfriend, failing end of year exams) [23], and 12 stressful independent events. Since most independent life events represent experiences necessarily common to both twins/siblings (e.g. parental divorce), they are unsuitable for twin analyses and were excluded. A subscale score for dependent stressful life events was calculated by summing the number of events reported on the relevant 12 items [23].

### 2.3 Statistical analyses

#### 2.3.1 Phenotypic analyses

We examined phenotypic associations between putative risk factors (maternal punitive discipline, paternal punitive discipline and stressful life events) and OCS at Times 1 and 2 using separate univariate linear regressions. Risk factors were then entered into a multiple linear regression model to determine whether they independently predicted OCS at both time-points. Longitudinal analyses involved a second multiple linear regression, testing whether putative risk factors independently predicted later OCS, while controlling for earlier OCS. The Time 2 measure of life events was used for longitudinal analyses in order to test whether events occurring *between* Time 1 and Time 2 predicted subsequent increases in OCS. Phenotypic analyses were conducted with the whole sample (i.e. all twins and siblings).

#### 2.3.2 MZ twin differences analyses

Phenotypic analyses were repeated using MZ twin difference scores, which effectively control for genetic and shared environmental factors. Since MZ twins are genetically identical and share their rearing environment, differences between members of an MZ twin pair must result from non-shared environmental influences. The MZ twin differences design is therefore a useful tool for isolating non-shared environmental effects. Relative MZ twin difference scores were calculated by randomly assigning twin members as Twin 1 and Twin 2, and subtracting the score of Twin 2 from that of Twin 1.

#### 2.3.3 Multivariate genetic modelling

To further explore the nature of the phenotypic associations between OCS and risk factors, and increase statistical power by including the DZ twins and siblings in the sample, we applied multivariate twin models to the data. The twin design compares the degree of phenotypic similarity between MZ twins, who share 100% of their genes, with DZ twins and FS, who shared 50% of their segregating genes on average [24]. Within-pair correlations for MZ twins are compared with within-pair correlations for DZ twins and FS in order to estimate the effects of: additive genetic factors (A); shared environment (C), defined as aspects of the environment resulting in phenotypic similarity between siblings; and non-shared environment (E), defined as environmental factors that give rise to phenotypic differences between siblings. Greater MZ compared to DZ/FS phenotypic similarity is attributed to genetic effects. Within-pair similarity that is not accounted for by genetic factors is attributed to shared environmental effects. Non-shared environmental effects, which are estimated from the within-pair differences between MZ twins, also include measurement error.

Putative risk factors which were significant, independent predictors of OCS in (a) cross-sectional and (b) longitudinal phenotypic analyses were included in the genetic models. A multivariate correlated factors solution was used for cross-sectional data. This model tests the extent to which phenotypic correlations between variables are due to correlations among the genetic and environmental factors that influence each of them. Thus, we could directly estimate the extent to which links between putative risk factors and OCS were mediated by genetic or environmental factors. We used a multivariate Cholesky decomposition for longitudinal data. In this model, genetic and environmental components for each variable are able to influence all subsequent variables, ordered according to their chronology. Thus, we could directly estimate the aetiology of the association between risk factors and OCS at Time 2, while controlling for the stable influence of aetiological factors from OCS at Time 1.

Phenotypic and MZ twin difference analyses were conducted using STATA version 14.1. Maternal and paternal punitive discipline were normally distributed but OCS and stressful life events at showed positive skew Time 1 and 2 and were therefore log-transformed (see Table A2 in Appendix A). All variables were standardised for ease of comparison. For phenotypic analyses, a robust cluster option was used to account for the non-independence of twins/siblings. All genetic modelling was conducted within R using OpenMx [25]. All analyses controlled for age and sex as is standard practice with twin data since failure to correct for age and sex effects can result in overestimation of twin correlations [26].

## 3 Results

### 3.1 Sample characteristics

Table 1 shows mean scores for study variables with respect to individual scores and MZ twin difference scores. For twin differences scores, the mean always approximates to zero since random assignment ensures that cases in which Twin 1 scores higher than Twin 2 are countered by cases in which the reverse is true. Importantly, the amount of variation in difference scores demonstrates the existence of differential experience. For example, the mean difference score for OCS at Time 1 was 0.06, showing that on average twins rate similar levels of OCS, but the *SD* was 3.31 meaning that approximately 32% of MZ twins differ by at least 3.31 points on OCS at Time 1.

**Table 1:** Descriptive statistics for study variables.

	Individual scores for whole sample			MZ twin difference scores		
	Mean	SD	Range	Mean	SD	Range
Time 1						
OCS	3.83	3.18	0 – 18	−0.06	3.31	−13 –15
Maternal punitive discipline	7.00	4.30	0 – 20	−0.07	3.76	−14 –13
Paternal punitive discipline	7.16	3.77	0 – 20	0.25	4.05	−15 –13
Stressful life events	1.22	1.41	0 – 9	−0.06	1.46	−6 – 5

Time 2						
OCS	3.08	2.96	0 – 18	−0.33	2.97	−10 – 9
Stressful life events	1.24	1.38	0 – 8	−0.12	1.30	-4 – 3

Note: OCS = obsessive-compulsive symptoms; MZ = monozygotic. Sample size ranges from n = 1471 to 2559 for raw scores and n = 206 to 344 for MZ twin difference scores. Summary statistics are presented on untransformed variables for comparison with other published samples.

### 3.2 Phenotypic analyses

Cross-sectional phenotypic associations are shown in the left-hand column of Table 2. Univariate linear regressions indicated that maternal punitive discipline, paternal punitive discipline and dependent stressful life events all showed positive associations with OCS at Time 1. Moreover, multiple regression revealed that all three factors were independently related to OCS.

**Table 2:** Results of linear regression analyses showing phenotypic associations between obsessive-compulsive symptoms and the putative environmental risk factors in the whole sample.

	Cross-sectional association (OCS at Time 1)				Longitudinal associations (OCS at Time 2)			
	β	*t*	*p*	R^2^	β	*t*	*p*	R^2^
**Univariate linear regressions**								
Factor 1: Maternal punitive discipline at Time 1	.19	8.34	<.001	.04	.15	5.52	<.001	.02
Factor 1: Paternal punitive discipline at Time 1	.16	6.76	<.001	.03	.12	4.19	<.001	.01
Factor 1: Dependent stressful life events at Time 1 / 2^1^	.24	11.65	<.001	.06	.23	8.90	<.001	.05

**Multiple linear regressions**								
Factor 1: Maternal punitive discipline at Time 1	.10	3.65	<.001	.08	.09	2.73	<.01	.07
Factor 2: Paternal punitive discipline at Time 1	.07	2.60	<.01		.05	1.39	.17	
Factor 3: Dependent stressful life events at Time 1 / 2^1^	.21	9.48	<.001		.21	7.77	<.001	

Factor 1: OCS at Time 1	N/A				.44	18.49	<.001	.25
Factor 2: Maternal punitive discipline at Time 1					.02	0.69	.49	
Factor 3: Paternal punitive discipline at Time 1					.02	0.53	.59	
Factor 4: Dependent stressful life events at Time 2					.16	6.64	<.001	

Note: OCS = obsessive-compulsive symptoms. Sample size ranges from n = 2239 to 2528 for cross-sectional analyses and from n = 1317 to 1483 for longitudinal analyses. Analyses corrected for relatedness in twin pairs using robust clustering. All analyses controlled for age and gender.

1 Dependent stressful life events reported at Time 1 were used for cross-sectional analyses. Dependent stressful life events reported at Time 2 were used for longitudinal analyses, in order to test whether life events that occurred *between* Time 1 and Time 2 predicted increased OCS.

Longitudinal phenotypic associations are shown in the right-hand column of Table 2. Again, univariate regressions demonstrated that maternal punitive discipline, paternal punitive discipline and dependent stressful life events were all associated with subsequent OCS (i.e. OCS at Time 2). However, multiple regression revealed that only maternal punitive discipline and stressful life events uniquely predicted variance in later OCS. A second multiple regression showed that when controlling for OCS at Time 1, only stressful life events predicted OCS at Time 2, suggesting that exposure to more stressful life events predicts the subsequent reporting of increased OCS.

### 3.3 MZ twin difference analyses

When controlling for genetic and shared environmental influences using MZ twin difference scores, neither paternal nor maternal punitive discipline were significantly associated with OCS at Time 1 (see left-hand column of Table 3). However, the association between stressful life events and OCS remained significant.

**Table 3:** Results of linear regression analyses predicting MZ twin differences in obsessive-compulsive symptoms from MZ twin differences in the putative environmental risk factors.

	Cross-sectional analyses (OCS at Time 1)				Longitudinal associations (OCS at Time 2)			
	β	*t*	*p*	R^2^	β	*t*	*p*	R^2^
**Univariate linear regressions**								
Factor 1: Maternal punitive discipline at Time 1	.06	1.17	.24	.00	.11	1.68	.10	.02
Factor 1: Paternal punitive discipline at Time 1	.09	1.63	.10	.01	.06	.82	.41	.01
Factor 1: Dependent stressful life events at Time 1 / 2^a^	.22	4.07	<.001	.05	.18	2.38	<.05	.03

**Multiple linear regressions**								
Factor 1: Maternal punitive discipline at Time 1	−.00	−.02	.99	.05	.10	1.22	.23	.06
Factor 2: Paternal punitive discipline at Time 1	.05	.71	.48		.01	.13	.90	
Factor 3: Dependent stressful life events at Time 1 / 2^a^	.20	3.40	<.01		.17	1.90	.06	

Factor 1: OCS at Time 1	N/A				.43	6.42	<.001	.26
Factor 2: Maternal punitive discipline at Time 1					.15	1.92	.06	
Factor 3: Paternal punitive discipline at Time 1					−.06	−.79	.43	
Factor 4: Dependent stressful life events at Time 2					.07	.87	.38	

Note: OCS = obsessive-compulsive symptoms. All variables are based on MZ twin difference scores. Sample size ranges from n = 280 to 341 for cross-sectional analyses and from n = 156 to 190 for longitudinal analyses. All analyses controlled for age and gender.

a Dependent stressful life events reported at Time 1 were used for cross-sectional analyses. Dependent stressful life events reported at Time 2 were used for longitudinal analyses, in order to test whether life events that occurred *between* Time 1 and Time 2 predicted increases in OCS.

Longitudinal analyses of MZ twin difference scores are shown in the right-hand column of Table 3. Univariate regressions indicated that only stressful life events were significantly associated with later OCS. This association did not remain significant when controlling for earlier OCS.

### 3.4 Multivariate genetic modelling

**Fig. 1:**
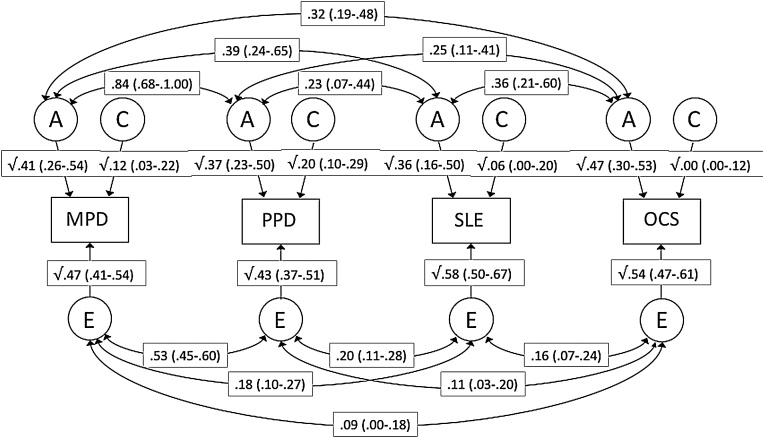
**Multivariate correlated factors model showing genetic and environmental influences on maternal punitive discipline, paternal punitive discipline, dependent stressful life events and obsessive-compulsive symptoms (all at Time 1).** Note: MPD = Maternal punitive discipline; PPD = Paternal punitive discipline; SLE = dependent stressful life events; OCS = obsessivecompulsive symptoms; A = additive genetic effects; E = non-shared environmental effects; values on single-headed arrows are standardized, squared path estimates; values on double-headed arrows are correlation coefficients; 95% confidence intervals in parentheses. Shared environmental correlations were fixed to zero.

Significant predictors from the multivariate regression of OCS at Time 1 (maternal punitive discipline, paternal punitive discipline and stressful life events) were included in a multivariate correlated factors model to establish the aetiologies of their cross-sectional links with OCS. An ACE model provided the best fit for the data. Since we observed universally non-significant contributions to covariance from C (rC), we tested a more parsimonious ACE model fixing rC to zero. This model provided the best fit to the data (see see Table A3 in Appendix A). Estimates from the model are shown in Fig. 1. Genetic effects accounted for the majority of the phenotypic covariance between OCS and each of maternal punitive discipline, paternal punitive discipline and stressful life events (75%, 65% and 63%, respectively). Importantly, the non-shared environmental effects were also significant, explaining 25%, 35% and 37% of the phenotypic association, respectively.

**Fig. 2:**
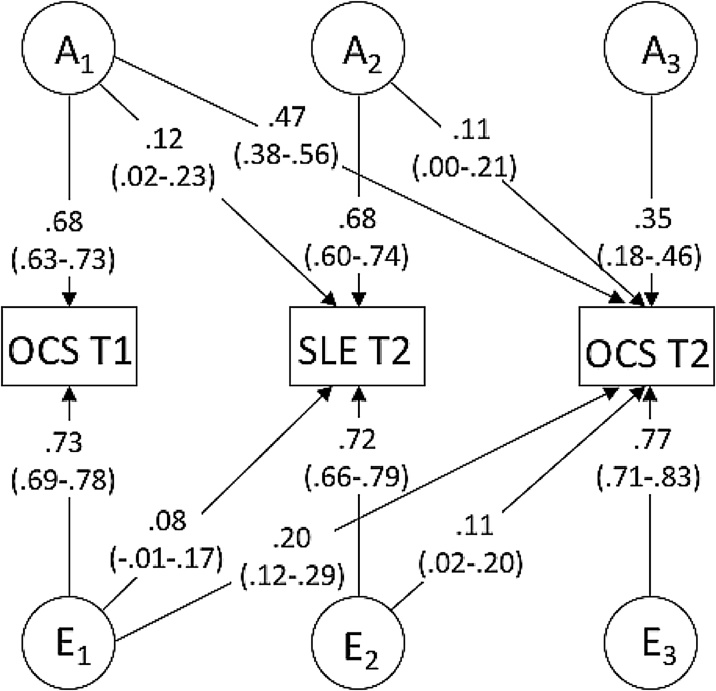
**Trivariate Cholesky decomposition showing genetic and environmental influences on obsessive-compulsive symptoms and stressful life events.** Note: OCS T1 = obsessive-compulsive symptoms at Time 1; SLE = dependent stressful life at Time 2; OCS T2 = obsessive-compulsive symptoms at Time 2; A = additive genetic effects; E = non-shared environmental effects; values are standardized, unsquared path estimates; 95% confidence intervals in parentheses Dependent stressful life events reported at Time 2 were used for longitudinal analyses, in order to test whether life events that occurred *between* Time 1 and Time 2 predicted increased OCS. The proportion of variance attributable to a specific variance component can be calculated by squaring the path estimate. This can be converted to a percentage by multiplying by 100. For example, the influence of E_2_ on OCS at Time 2 is .11^2^ x 100 = 1.21%. The environmental contribution to the association between stressful life events and OCS at Time 2 can be calculated by tracing the path between SLE T2 and OCS T2 via E_2_ (.72 × .11) and dividing it by the combination of the paths between SLE T2 and OCS T2 via A_2_ and E_2_ (.68 × .11 + .71 × .11).

Phenotypic analyses indicated that stressful life events predicted OCS at Time 2, after controlling for OCS at Time 1. A trivariate Cholesky decomposition was used to determine the aetiology of this association. An AE model provided the best fit for the data (see Table A4 in Appendix A). Estimates from the model are shown in Fig. 2. Path tracing showed that the unique phenotypic association between stressful life events and later OCS, after controlling for OCS at Time 1, was influenced by both genetic and non-shared environmental factors (48% and 52%, respectively). After accounting for genetic and non-shared environmental influences on OCS at Time 1, the additional non-shared environment factors associated with stressful life events at Time 1 accounted for 1.21% of the variance in OCS at Time 2 (i.e. 0.11^2^ x 100). The additional genetic influences also accounted for 1.21% of the variance in later OCS.

## 4 Discussion

This is the first study to attempt to isolate environmental effects on OCS using a longitudinal, genetically-informative design. Specifically, we aimed to clarify the extent to which punitive parenting and dependent stressful life events predict the development of OCS in adolescence, controlling for genetic and shared environmental influences.

Regarding our first aim, as hypothesised maternal punitive discipline, paternal punitive discipline and stressful life events all showed significant, independent phenotypic associations with adolescent OCS at Time 1. This is consistent with previous findings that negative parenting [3,4] and stressful life events [13,14] are related to OCD. With respect to longitudinal associations, contrary to our hypothesis we found that only stressful life events predicted later OCS when controlling for earlier OCS. In other words, greater levels of self-reported stressful life events, but not punitive parenting, predicted increases in OCS during adolescence.

Our second aim was to test whether associations between the putative environmental risk factors and OCS remained significant after controlling for genetic and shared environmental influences. As hypothesised, our MZ twin difference analyses showed that stressful life events were positively associated with OCS at Time 1. This finding is consistent with previous studies in adults [13,14], and demonstrates that the association between OCS and life events is independent of familial confounds. MZ twin difference analyses also provided partial evidence for a longitudinal association between stressful life events and OCS, controlling for familial effects. This association was significant in the univariate regression but not the multiple regressions, which could reflect reduced statistical power.

Contrary to our hypothesis, univariate regressions of MZ twin difference scores indicated that neither maternal nor paternal punitive discipline were significantly associated with OCS either cross-sectionally or longitudinally when accounting for familial confounding. This could imply that the phenotypic relationship between punitive parenting and OCS is largely driven by genetic and/or shared environmental effects. Alternatively, these analyses may have been underpowered to detect modest effects given the reduced sample size (only MZ twins). We therefore further explored genetic and environmental influences on phenotypic associations of interest using genetic models.

Results from the genetic modelling indicated that cross-sectional phenotypic associations between the three putative environmental risk factors and OCS are mediated by both common genetic *and* non-shared environmental influences. Thus, as hypothesised, we found evidence consistent with a true environmental association of OCS with punitive parenting and dependent stressful life events. Similarly, in line with our hypothesis, the longitudinal genetic model indicated that the unique phenotypic association between stressful life events and later OCS (i.e. controlling for earlier OCS) is accounted for by both genetic and environmental factors, in roughly equal proportions. In this stringent test, stressful life events accounted for just over 1% of the variance in later OCS, independent of genetic confounds and after controlling for earlier OCS. While the magnitude of the effect is small, this is not surprising given that this analysis accounted for the stable and therefore more reliable variance. Furthermore, the genetic models include measurement error in the E term, potentially attenuating our estimates of non-shared environmental effects. Nevertheless, our finding is in keeping with estimates found in previous genetically informed studies of concurrent associations between life events and OCS [13]. To our knowledge, this is the first study to demonstrate that stressful life events prospectively predict the development of new OCS, when controlling for genetic effects. This finding supports the hypothesis that such experiences play a causal role in the development of OCS. Future studies should further test causality using established epidemiological criteria [27]. Additionally, further research is needed to examine the mechanisms underlying the association between stressful life events and OCS. Cognitive behavioural models propose that the impact of life events on OCS is moderated by cognitive biases [28]. This could be tested in longitudinal, genetically-informative studies using self-report measures and/or behavioural assessments of information-processing biases.

The current findings have important implications. First, higher levels of self-reported dependent stressful life events predicted subsequent increases OCS during adolescence at a phenotypic level. Thus, stressful life events could be a clinical marker for identifying individuals at risk of developing OCD, in conjunction with other known risk factors (e.g. family history of OCD), enhancing early intervention and long-term outcomes [29]. Second, the longitudinal association between stressful life events and OCS exists above and beyond common genetic influences, consistent with the notion that such experiences represent an environmental risk for the development of OCD. Although further research is needed to understand the mechanisms underpinning this relationship, stressful life events could be an important target for prevention programs. Lastly, we did not find evidence for punitive parenting being a risk factor for OCS, which implies that modifying such parenting behaviours is unlikely to be an effective OCD prevention strategy during adolescence.

The current study has several strengths including the prospective, genetically-informative design and inclusion of fathers, who are often overlooked in studies of parenting [30]. Nevertheless, results should be considered in the context of some limitations. First, only punitive parenting was assessed and findings may not generalise to other parenting practices (e.g. overprotective parenting). Second, our measure of stressful life events relied on retrospective reports of the previous 12 months. However, recall bias is likely to be lower compared to previous studies which retrospectively assessed life events across the life span (e.g. [13,14]. Third, our study focussed on OCS and it cannot be assumed that the results would generalise to diagnosable OCD, although empirical data indicate that OCD is a dimensional construct [31]. Fourth, this study only included adolescent-report measures. Reliance on a single informant may have inflated associations and future studies should utilise multiple-informant measures. Fifth, we found that participant drop out was predicted by a range of variables including OCS at Time 1. The magnitude of effects were small but reached statistical significant due to the large sample size. Although selective attrition may have reduced the overall variance at Time 2, it is unlikely to have substantially influenced our estimates of associations [32].

In summary, the present study suggests that dependent stressful life events, but not punitive parenting, predict the subsequent reporting of increased OCS during adolescence. Furthermore, this association exists independent of genetic effects. While these findings are consistent with the notion that stressful life events play a causal role in the development of OCD, further research is needed to establish the mechanisms underlying this association.

## Funding

Waves 1–3 were funded by the W T Grant Foundation, the University of London Central Research fund and a Medical Research Council (MRC) Training Fellowship (G81/343) and Career Development Award (G120/635) to Thalia C. Eley. Georgina Krebs is funded by an MRC Clinical Research Training Fellowship (MR/N001400/1). Laurie Hannigan is supported by a 1 + 3 Ph.D. studentship from the UK Economic and Social Research Council (ESRC). This study presents independent research part-funded by the National Institute for Health Research (NIHR) Biomedical Research Centre at South London and Maudsley NHS Foundation Trust and King’s College London. The views expressed are those of the authors and not necessarily those of the NHS, the NIHR or the Department of Health.

## Declarations of interest

None.

## Figures and Tables

**Table A1:** Results of logistic regression showing variables predicting participant drop-out between Time 1 and Time 2.

Variable	Odds Ratio (95% confidence intervals)	*z*	*p*
Sex	1.48 (1.21 - 1.81)	3.93	<.001
Age	0.90 (0.84 - 0.96)	−3.22	<.01
Socioeconomic status (parental education level)	1.06 (1.01 - 1.12)	2.25	<.05
OCS at Time 1	0.97 (0.94 - 1.00)	2.43	<.05
Maternal punitive discipline at Time 1	0.97 (0.94 - 1.00)	−1.83	.067
Paternal punitive discipline at Time 1	0.99 (0.96 - 1.01)	−1.00	.316
SLE at Time 1	0.48 (0.32 - 0.72)	−3.52	<.001

Note: OCS = obsessive-compulsive symptoms; SLE = stressful life events.

**Table A2:** Skewness of variables before and after log transformation.

Variable	Skewness of untransformed variable	Skewness of log transformed variable
OCS at Time 1	1.19	.34
OCS at Time 2	1.55	.12
SLE at Time 1	1.39	.35
SLE at Time 2	1.22	.29
Maternal punitive discipline at Time 1	.53	N/A
Paternal punitive discipline at Time 1	.55	N/A

Note: OCS = obsessive-compulsive symptoms; SLE = stressful life events.

**Table A3:** Fit comparisons for mulitvariate correlated factors models.

Base model	Comparison model	−2LL	df	diff LL	diff df	*p*	AIC
Saturated model	N/A	25313.92	9553	N/A	N/A	N/A	6207.92
Saturated model	ACE model	25375.67	9607	61.75	54	0.22	4181.95
ACE model	**ACE model (No rC)**	25,379**.83**	**9613**	**4.16**	**6**	**0.66**	**6153.83**
ACE model	AE model	25409.94	9617	34.27	10	<.001	6175.94

Note: -2LL = minus twice the log likelihood; AIC = Akaike’s information criterion; A = additive genetic influence; C = shared environmental influences; E = non-shared environmental influences. Bold typeface indicates the selected model.

**Table A4:** Fit comparisons for trivariate Cholesky decomposition models.

Base model	Comparison model	−2LL	df	diff LL	diff df	*p*	AIC
Saturated model	N/A	14869.59	5439	N/A	N/A	N/A	3991.59
Saturated model	ACE model	14906.49	5472	36.9	33	0.56	3962.49
ACE model	**AE model**	14906**.67**	**5478**	**0.18**	**6**	**0.99**	**6480.98**

Note: -2LL = minus twice the log likelihood; AIC = Akaike’s information criterion; A = additive genetic influence; C = shared environmental influences; E = non-shared environmental influences. Bold typeface indicates the selected model.
